# The Role of Sleep Banking in Reducing Cognitive and Motor Impairments from Subsequent Sleep Restriction: A Narrative Review

**DOI:** 10.3390/clockssleep8010008

**Published:** 2026-02-23

**Authors:** Alen Juginović, Laura Rodman

**Affiliations:** 1 Department of Neurobiology, Harvard Medical School, 220 Longwood Avenue, Boston, MA 02115, USA; 2 Veterinary Faculty, University of Zagreb, Heinzelova 55, 10000 Zagreb, Croatia

**Keywords:** sleep banking, sleep extension, sleep deprivation, cognitive performance, circadian rhythm, homeostatic sleep regulation

## Abstract

Sleep banking, i.e., preemptively obtaining extra sleep prior to anticipated sleep loss, has been proposed as a strategy to reduce the cognitive and physiological consequences of sleep deprivation. However, our understanding remains incomplete regarding the effectiveness of preemptive sleep extension in enhancing resilience to sleep loss. A comprehensive literature search was conducted using PubMed, MEDLINE, and Embase for studies published between 2004 and 2025. Following a comprehensive literature search, we identified 12 studies meeting the inclusion criteria—seven primary experimental trials comprising approximately 140 participants, predominantly healthy young adults aged 18–39 years. We evaluated the effects of sleep banking on cognitive performance, mood, physiological parameters, and real-world outcomes. Included studies encompassed experimental laboratory trials, observational research, and field studies in occupational and athletic settings. Although the number of studies on sleep banking remains limited, experimental evidence demonstrates that preemptive sleep extension improves objective alertness and vigilance during subsequent sleep restriction or total sleep deprivation. Individuals who obtained additional sleep exhibited fewer attentional lapses, faster reaction times, and improved mood, although subjective sleepiness often remained high. Preliminary field evidence suggests that preemptive sleep extension enhances workplace safety, reduces errors, and improves sustained attention in shift workers. In athletic contexts, sleep banking has been associated with improved physical endurance and reaction speed. Importantly, this review primarily addresses the homeostatic dimension of sleep regulation (Process S); circadian factors (Process C), including chronotype, social jetlag, and circadian timing of sleep extension and testing, were not systematically addressed in the included studies and represent important limitations of the current evidence base. Overall, sleep banking appears to be a viable strategy for enhancing resilience to acute sleep loss. It confers measurable benefits in performance, cognitive function, and physiological markers, supporting its application in high-demand occupations and competitive environments. Although it does not fully eliminate subjective fatigue, sleep banking may serve as a valuable complement to other fatigue mitigation strategies for anticipated short-term sleep loss.

## 1. What Was Known

Prior research has shown that acute sleep deprivation impairs vigilance, reaction time, mood, and physical performance. A small body of work and anecdotal evidence suggested that extending sleep before sleep loss may improve baseline alertness and reduce attentional lapses. However, the effectiveness, scope, and mechanisms of this “sleep banking” strategy had not been comprehensively synthesized, leaving uncertainty about its true role in enhancing resilience to sleep loss.

## 2. What This Study Adds

This narrative review synthesizes the fragmented evidence on preemptive sleep extension and provides the first integrated evaluation of its cognitive, physiological, and real-world effects. By comparing findings across laboratory trials, athletic contexts, and occupational settings, the review clarifies where sleep banking consistently offers measurable benefits and where its effects remain limited or uncertain. We explicitly situate sleep banking within the two-process model of sleep regulation, acknowledging that current evidence primarily addresses homeostatic mechanisms while circadian contributions remain understudied. This work establishes a clearer foundation for understanding sleep banking as a practical strategy for enhancing resilience to short-term sleep loss.

## 3. Introduction

Sleep is essential for a wide range of physiological and psychological processes, including memory consolidation, immune function, metabolic regulation, cardiovascular health, and emotional stability [1,2,3,4,5]. Chronic sleep insufficiency has been associated with elevated risks of obesity, type 2 diabetes, hypertension, and neurodegenerative diseases, as well as with impaired decision-making, reduced productivity, and increased all-cause mortality [5,6,7,8]. However, modern lifestyles and occupational demands often curtail sleep, leading to chronic “sleep debt” and associated impairments in health and performance [9,10]. On average, adults report sleeping around 6.8 h on weeknights, less than the 7–9 h generally considered necessary for optimal alertness [11]. Often the first symptoms of sleep loss include a decline in cognitive function, mood, and an increased risk of accidents and errors, all of which are undesirable for work productivity and safety [12]. A significant challenge is that individuals often underestimate the extent of their impairment; subjective feelings of sleepiness do not consistently parallel objective performance deficits [13]. Given the high prevalence of insufficient sleep in modern societies driven by shift work, screen use, social demands, and work overload, developing effective strategies to protect against the adverse effects of acute sleep loss is a pressing public health challenge.

Understanding sleep banking requires situating it within the broader framework of sleep–wake regulation. The two-process model posits that sleep timing and intensity are governed by two interacting processes: a homeostatic process (Process S) that accumulates sleep pressure during wakefulness and dissipates during sleep, and a circadian process (Process C) that modulates sleep propensity according to an approximately 24 h biological rhythm [14]. Sleep banking, as conceptualized in the literature, primarily targets Process S by reducing accumulated sleep pressure before anticipated sleep loss. However, modern societies are also characterized by widespread circadian disruption, including social jetlag (the discrepancy between biological and social clocks) and chronic circadian misalignment in shift workers. These circadian factors independently affect cognitive performance, mood, and health outcomes. Importantly, the method by which sleep is extended, earlier bedtimes versus later wake times, may differentially affect circadian phase position, a consideration that has received limited attention in sleep banking research.

Traditionally, efforts to mitigate the effects of insufficient sleep have focused on recovery sleep (“repaying” sleep debt after sleep loss) or on acute countermeasures such as caffeine or naps. A complementary and more proactive strategy is sleep banking, which involves obtaining extra sleep in advance of an anticipated period of sleep restriction or deprivation [15]. The rationale is analogous to saving energy in reserve: by extending sleep duration before a sleepless challenge, one might “store” benefits that buffer against fatigue. The concept of sleep banking emerged from experimental observations that extended sleep can enhance alertness beyond an individual’s habitual baseline. Multiple studies have shown that when individuals are given the opportunity to sleep longer than usual, they experience reduced sleepiness and improved performance and mood [16,17]. This raised the intriguing possibility that sleep not only serves to discharge prior sleep need but can also provide a reserve capacity that might be drawn upon during subsequent periods of wakefulness.

One of the first direct pieces of evidence for sleep banking was reported by Rupp et al., in which healthy adults who obtained extra sleep (10 h in bed per night for one week) showed greater resilience to cognitive impairment during a following week of severe sleep restriction (3 h sleep per night), compared to those who had maintained a normal 7 h sleep schedule [11]. This finding spurred further investigations into the extent and mechanisms by which preemptive sleep extension might offset the effects of sleep loss. Given the potential importance of this strategy for public health and safety, we review the current evidence on whether sleep banking can indeed be beneficial. We examine its effects on cognitive and psychomotor performance, mood, and physiological parameters, as well as any documented health or safety outcomes. We also consider real-world applications, such as in shift workers or athletes, and discuss underlying mechanisms. Through this narrative review, we aim to clarify the efficacy of sleep banking and its role as an acute fatigue mitigation tool, while acknowledging important limitations related to circadian biology that warrant future investigation.

## 4. Results

### 4.1. Effects on Cognitive Performance and Alertness

**Figure 1:**
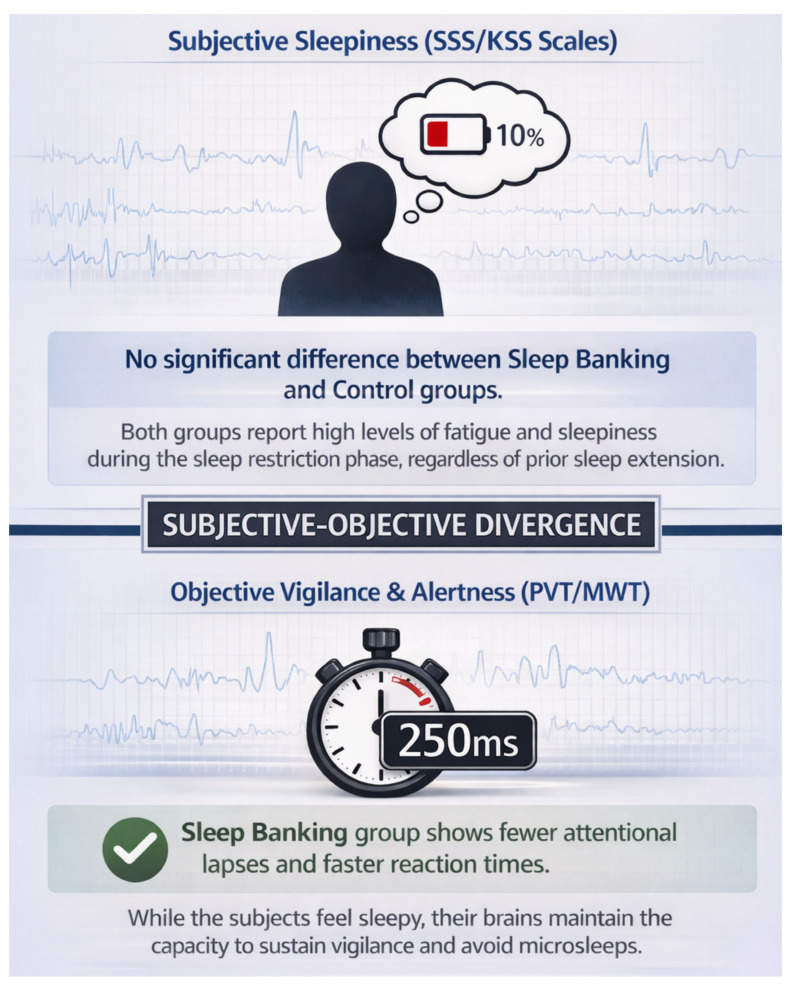
Subjective sleepiness versus objective vigilance: effects of sleep banking (AI-generated figure created with ChatGPT, OpenAI, version GPT-4.1, accessed 20 December 2025).

Multiple controlled experiments have demonstrated that sleep banking improves objective measures of alertness and vigilance during subsequent sleep loss. In the study by Rupp et al., participants who obtained ~1–2 h of extra sleep per night for one week showed significantly better psychomotor vigilance during a following week of sleep restriction (3 h time in bed nightly) compared to control subjects who maintained 7 h time in bed [11]. Specifically, the sleep-banked group experienced fewer lapses on the psychomotor vigilance task (PVT; mean ~6 lapses vs. ~12 in controls by day 7 of restriction) and could maintain wakefulness longer on the maintenance of wakefulness test (MWT) throughout the sleep-restriction period. Similarly, a randomized crossover trial by Arnal et al. found that six nights of sleep extension (~9.8 h in bed per night) markedly limited the deterioration in sustained attention during ~38 h of total sleep deprivation [16]. Participants who had banked sleep exhibited fewer PVT lapses (median ~8 vs. ~16 in controls during night-time wakefulness) and fewer involuntary microsleeps (brief EEG-confirmed sleep episodes) during overnight wakefulness, in contrast to those who slept habitually (8 h time in bed) before deprivation [16]. Notably, the study reported that these benefits persisted into the recovery period: even after one night of recovery sleep, the group that had extended sleep still showed faster reaction times (mean ~258 ms vs. ~280 ms in controls) and fewer lapses than the control group, indicating a carry-over performance benefit from sleep banking. (Figure 1. Subjective sleepiness versus objective vigilance: effects of sleep banking (AI-generated figure created with ChatGPT4.1, OpenAI, version GPT-4.1, accessed 20 December 2025)).

However, the impact of sleep banking on higher-order executive functions is less clear. Re-analysis of the Arnal et al., 2015 data showed that prior sleep extension was not effective in mitigating deficits in tasks requiring response inhibition (a Go-No Go reaction task) or working memory (a 2-Back task) during total sleep deprivation [16,18]. This suggests a dissociation in which basic vigilance is more readily buffered by sleep banking than more complex, prefrontal cortex-dependent cognitive processes. In contrast, a study by Ritland et al. on military tactical athletes did find a significant improvement in one aspect of executive function (cognitive flexibility on the Trail Making Test Part B times by ~22% compared to baseline) after four nights of sleep extension, highlighting that some executive benefits can occur depending on the task and context [19]. It is important to note, however, that this study did not include a subsequent sleep deprivation phase, so it demonstrates cognitive benefits of sleep extension per se rather than protection against subsequent sleep loss. Taken together, the evidence, although limited, supports that banking sleep confers measurable resilience to the cognitive slowing and attentional failures induced by short-term sleep loss.

Despite these improvements in objective performance, subjective sleepiness often remains high in individuals who bank sleep. In Rupp et al.’s study, for example, participants’ ratings of sleepiness (e.g., on the Stanford Sleepiness Scale) did not differ significantly between the extended-sleep group and the normal-sleep group during the restriction phase [11]. Likewise, Arnal et al. found that Karolinska Sleepiness Scale scores rose substantially during total sleep deprivation and were not significantly lower in the sleep extension condition [16]. In other words, those who banked sleep still felt as sleepy as those who had not, even though their performance was objectively better. This dissociation between subjective and objective measures suggests that extra sleep may improve the brain’s capacity to sustain vigilance without lapsing, but it does not fully alleviate the feeling of fatigue. This dissociation may partly reflect the independent contribution of circadian alerting signals, which modulate subjective alertness according to time of day regardless of prior sleep history. Nonetheless, mood and sense of well-being may benefit: one laboratory study noted improvements in mood (including reduced irritability and increased positivity) after a week of extended sleep [20]. In healthy young adults, extended sleep led to higher daytime alertness and better mood as compared to habitual sleep schedules [20]. These mood benefits were also observed in the context of sleep banking before sleep loss. Overall, cognitive testing and self-reported outcomes indicate that banking sleep can bolster alertness and help maintain mood at a more stable level during ensuing wakefulness, even if one still perceives some sleepiness.

### 4.2. Athletic and Physical Performance Outcomes

Sleep banking has demonstrated notable benefits for physical performance and endurance. One study examined neuromuscular performance in a crossover trial where subjects either extended their sleep (9.8 h time in bed) or maintained normal sleep (8 h time in bed) for six nights, followed by one night of total sleep deprivation [21]. After sleep loss, the extended-sleep condition was associated with a longer time to exhaustion in a sustained isometric exercise (a leg muscle endurance task) compared to the normal-sleep condition [21]. In fact, even at baseline (prior to deprivation), those who had extended sleep showed a modest (~4%) increase in endurance time, and this performance advantage was preserved (~8% longer endurance) after the night of no sleep [21]. Ratings of perceived exertion during exercise were also lower following extended sleep, suggesting participants felt the exercise was easier after banking sleep [21]. Notably, these improvements in endurance occurred without differences in maximal muscle strength or voluntary activation, indicating that the benefit was likely due to central factors (reduced central fatigue or discomfort) rather than changes in muscle physiology [21]. The authors concluded that banked sleep improves physical resilience to the stress of sleep deprivation, likely by blunting the subjective fatigue that can hasten exhaustion [21].

In athletes, extending sleep has yielded significant performance gains. In a well-known study on collegiate basketball players, Mah et al. had players increase their sleep duration substantially (aiming for ~10 h in bed each night) for 5–7 weeks during their season [22]. The results were striking: after this sleep extension period, the athletes ran a timed sprint faster (improving from 16.2 ± 0.6 s to 15.5 ± 0.5 s over a ~86 m sprint; *p* < 0.001) and significantly improved their shooting accuracy, with free-throw and 3-point shot success rates each increasing by about 9% [22]. Objective reaction times on the PVT also improved, and players reported less daytime sleepiness and improved mood, including higher vigor and reduced fatigue on standardized mood scales [22]. It is important to note that this study examined sleep extension without subsequent sleep deprivation or restriction. Therefore, while it demonstrates that extended sleep improves athletic performance from baseline—suggesting potential “ceiling” benefits that could buffer against later sleep loss—it does not directly test the sleep banking paradigm. The observed improvements may reflect resolution of pre-existing chronic sleep debt rather than creation of a true performance reserve. Other athletic populations (e.g., runners and tennis players) have similarly shown better sprint times, reaction speed, and endurance when prioritizing extra sleep in training [23,24]. Moreover, even in physically demanding military settings, extending sleep has proven beneficial: a trial with military trainees (sometimes termed “tactical athletes”) found that four nights of sleep extension led to improved physical performance, including significantly longer standing broad jump distances, and that this benefit persisted for four days after returning to a normal sleep schedule [19]. This study, while demonstrating persistent benefits of sleep extension, also did not include a formal sleep deprivation phase. Taken together, the benefits of sleep banking translate into real-world performance improvements across both athletic and occupational physical tasks, though direct evidence for physical performance protection during subsequent sleep loss remains limited to the Arnal et al., 2016 study [21].

### 4.3. Physiological and Health-Related Outcomes

Sleep banking primarily aims to improve neurobehavioral functioning, but its effects on underlying physiological markers have also been investigated, albeit to a limited extent. One key question is whether extending sleep alters the body’s hormonal or homeostatic responses to subsequent sleep deprivation. In a controlled crossover study, Chennaoui and colleagues measured endocrine responses with and without prior sleep extension [25]. They found that 24 h of continuous wakefulness led to significant drops in morning testosterone and cortisol (stress hormone), as expected, and sleep extension did not prevent these hormonal changes [25]. In other words, despite having a week of >9 h bedtimes, participants still experienced the usual suppression of these hormones after one day of no sleep, to a similar degree as when they had no extra sleep [25]. This suggests that the benefits of sleep banking are not primarily mediated by baseline levels of those particular hormones. However, interpretation of these hormonal findings requires consideration of circadian rhythmicity: testosterone and cortisol exhibit robust circadian patterns, with peak levels in the early morning. The observed “suppression” following sleep deprivation may partly reflect circadian phase-dependent sampling rather than, or in addition to, true physiological suppression from sleep loss per se. Interestingly, that same study noted an effect on prolactin: baseline morning prolactin concentrations were slightly lower after the sleep-extension week than after a normal sleep week, and this difference persisted even after one night of recovery sleep [25]. The clinical significance of lower prolactin is unclear, but it hints that extended sleep may subtly alter certain aspects of neuroendocrine function (prolactin is associated with sleep regulation and immune function).

Sleep extension also influences metabolic and neurotrophic factors. Chennaoui et al. reported that a week of sleep extension significantly increased insulin-like growth factor 1 (IGF-1) levels in healthy young men, both at baseline and during a subsequent period of total sleep deprivation [25]. IGF-1 is a hormone involved in neuroplasticity, metabolism, and tissue repair; elevated IGF-1 levels may reflect enhanced restorative capacity or improved metabolic support for neuronal function in response to extended sleep. Like other hormones, IGF-1 exhibits diurnal variation, though its rhythm is less pronounced than cortisol or testosterone. The timing of blood sampling relative to sleep–wake and circadian phase should be considered when interpreting these findings.

In another study, after a week of extended nights, participants showed longer latency to fall asleep in the daytime (on the Multiple Sleep Latency Test) compared to their baseline, potentially indicating reduced physiologic sleep drive [16]. Additionally, polysomnographic data show that when people are given an extended sleep opportunity, they not only sleep longer overall but also obtain more of the deeper stages of sleep than usual [16]. In the Arnal et al. trial, total sleep time increased by ~1–1.2 h per night with extension, and this increase was distributed across all sleep stages: stage 1, stage 2, REM, and even a modest ~10% increase in stage 3 slow-wave sleep [16]. These findings suggest that sleep banking may enhance overall sleep quality and restorative depth, which in turn could underlie the observed performance benefits.

Beyond laboratory measures, extending sleep may confer some metabolic health advantages. In one four-week study, adults who were habitual short sleepers and increased their nightly time in bed subsequently reduced their intake of free (added) sugars in the diet by ~10 g/day (~38% reduction), even though other measures like body weight did not change over that short term [26]. This finding suggests that improving sleep duration could positively influence dietary choices and metabolic risk factors, although more research is needed to confirm long-term health implications.

### 4.4. Circadian Considerations and Limitations

The evidence reviewed above primarily addresses the homeostatic dimension of sleep–wake regulation (Process S in the two-process model), with limited consideration of circadian factors (Process C). This represents an important limitation of the current literature that warrants discussion. According to the two-process model of sleep regulation [14], sleep timing, duration, and quality are determined by the interaction of homeostatic sleep pressure (which accumulates during wakefulness and dissipates during sleep) and circadian rhythmicity (an approximately 24 h biological clock that modulates alertness and sleep propensity independently of prior sleep history). Sleep banking, as currently conceptualized, targets Process S: by reducing accumulated homeostatic pressure through extended sleep, individuals may enter a period of sleep loss with greater “reserve capacity.” However, Process C continues to modulate performance according to circadian phase, leading to well-documented time-of-day effects on vigilance, reaction time, and cognitive performance that operate independently of sleep history.

Most laboratory studies of sleep banking have tested participants at fixed clock times without systematically accounting for circadian phase. The observed benefits of sleep banking during overnight wakefulness [16] may therefore reflect an interaction between reduced homeostatic pressure and preserved circadian alerting signals, rather than purely homeostatic effects. Studies have not systematically examined whether sleep banking benefits are consistent across different circadian phases. A critical but under-addressed question is whether sleep extension is achieved through earlier bedtimes, later wake times, or both, as these approaches have different implications for circadian phase. Later wake times could delay circadian phase, particularly when morning light exposure is avoided, whereas earlier bedtimes might phase-advance the circadian system if coupled with earlier morning light exposure. Symmetric extension involving both earlier bedtimes and later wake times might have minimal net phase effects. Most sleep banking studies have employed laboratory protocols with controlled light exposure, minimizing unintended circadian phase shifts; however, in real-world applications, the method of extension could substantially affect both the quality of extended sleep and subsequent performance.

Individual differences in chronotype (morningness-eveningness preference) have not been systematically examined in sleep banking studies. Evening chronotypes may find it easier to extend sleep by sleeping later in the morning, while morning chronotypes may more readily extend by going to bed earlier. Furthermore, chronotype influences the timing of peak performance: evening types may be more resilient to overnight wakefulness due to later circadian phase [14], yet whether sleep banking efficacy differs by chronotype remains unknown. Many participants in sleep banking studies, particularly young adults, likely experience chronic social jetlag, a discrepancy between biological and social clocks that leads to sleep curtailment on work/school days and compensatory sleep extension on free days. In this context, the “control” condition of habitual sleep may itself represent a state of partial sleep restriction and circadian misalignment, and the observed benefits of sleep extension could therefore partly reflect resolution of this chronic circadian disruption rather than, or in addition to, creation of a true sleep “reserve.” This interpretation is supported by the observation that benefits of sleep banking are most pronounced in populations with shorter habitual sleep durations. Taken together, the current evidence cannot fully disentangle homeostatic from circadian contributions to sleep banking efficacy, and the interaction between sleep extension protocols and circadian biology remains an important gap in the literature.

## 5. Discussion

The collective findings from experimental and field studies (despite the limited number) provide convergent evidence that sleep banking may be an effective strategy to enhance human resilience to short-term sleep loss. By obtaining extra sleep in advance, individuals can attenuate the cognitive slowing, attentional lapses, and fatigue-related symptoms that typically accompany sleep deprivation. Our review found improvements across a range of outcomes, from simple reaction time and vigilant attention to athletic sprint performance, in subjects who had extended their sleep prior to sleep restriction [16,22,23]. These benefits of sleep banking, observed in both total sleep deprivation and partial sleep restriction paradigms, substantiate the concept that the brain and body can be “preloaded” with sleep to better withstand a subsequent deficit. In essence, getting sufficient sleep beforehand can be viewed as building a buffer against homeostatic sleep pressure, allowing individuals to maintain higher levels of functioning for longer into a period of wakefulness than they otherwise could.

### 5.1. Interpretation: Sleep Debt Repayment vs. True Reserve

An ongoing question is whether sleep banking’s benefits primarily reflect the repayment of an existing sleep debt or the creation of a true performance reserve beyond normal sleep needs. In many studies, the “normal sleep” control groups may themselves be chronically slightly sleep-restricted, suggesting that the gains seen in the extended-sleep groups partly represent recovery of baseline function. This is a critical interpretive issue: if participants habitually sleep 6–6.5 h (as is common among young adults and athletes), then a “control” condition of 7–8 h time in bed may not represent truly adequate sleep, and an “extension” condition of 10 h may primarily be restoring function to biological baseline rather than creating excess reserve. However, certain observations, such as faster recovery and sustained performance advantages even after the sleep deprivation phase, hint at a modest reserve effect [23]. It is likely that both mechanisms contribute: extra preemptive sleep both eliminates pre-existing sleep debt and potentially provides an additional cushion of resilience, especially when baseline sleep was suboptimal. Future studies should carefully characterize participants’ baseline sleep debt using objective measures (e.g., MSLT at baseline, sleep diary + actigraphy for 2+ weeks prior) to distinguish these mechanisms.

### 5.2. Homeostatic Mechanisms

The physiological underpinnings of sleep banking’s efficacy appear to center on homeostatic mechanisms and neuroplastic changes. From a homeostatic sleep regulation perspective, entering a period of sleep loss well-rested means starting with a lower level of accumulated sleep drive (Process S in the two-process model) [14]. Extended sleep opportunities enable a more complete dissipation of sleep pressure. For example, Arnal et al. demonstrated that multiple nights of 10 h time in bed led to significantly longer MSLT latencies (i.e., lower sleep propensity) at baseline compared to normal sleep. In other words, beginning a sleepless period in a well-rested, low sleep-pressure state delays the point at which cognitive function deteriorates during prolonged wakefulness [16]. There is also an allostatic perspective: by reducing the body’s cumulative stress burden (“allostatic load”) through extra sleep, an individual may increase their physiological capacity to cope with the subsequent strain of sleep deprivation. By proactively minimizing the wear-and-tear from prior insufficient sleep, one enters the sleep loss period with more robust neuroendocrine stability, potentially moderating stress responses.

### 5.3. Circadian Limitations of Current Evidence

While the homeostatic framework provides a compelling explanation for sleep banking benefits, it is incomplete without consideration of circadian factors. As discussed in other sections, the circadian system independently modulates alertness, performance, and physiological function according to a ~24 h rhythm, and several important limitations apply to the current evidence base. Circadian phase was not measured or controlled in most studies, leaving uncertainty about whether extended sleep altered circadian timing or whether benefits were consistent across different circadian phases. Hormonal findings also require circadian interpretation; the observation that cortisol and testosterone remained suppressed after sleep deprivation despite prior extension [21] does not definitively indicate that sleep banking failed to protect these systems, as it may simply reflect that circadian-phase-dependent sampling windows differed between conditions or that circadian modulation of hormone secretion is independent of homeostatic status. Furthermore, real-world applications involve circadian challenges that were not present in laboratory studies: shift workers, athletes competing across time zones, and military personnel on operational schedules face combined homeostatic and circadian disruption, and the laboratory evidence for sleep banking may not translate directly to these contexts where circadian misalignment compounds sleep loss. Finally, whether individuals extend sleep by going to bed earlier or waking up later has different implications for circadian physiology that have not been systematically studied.

**Figure 2:**
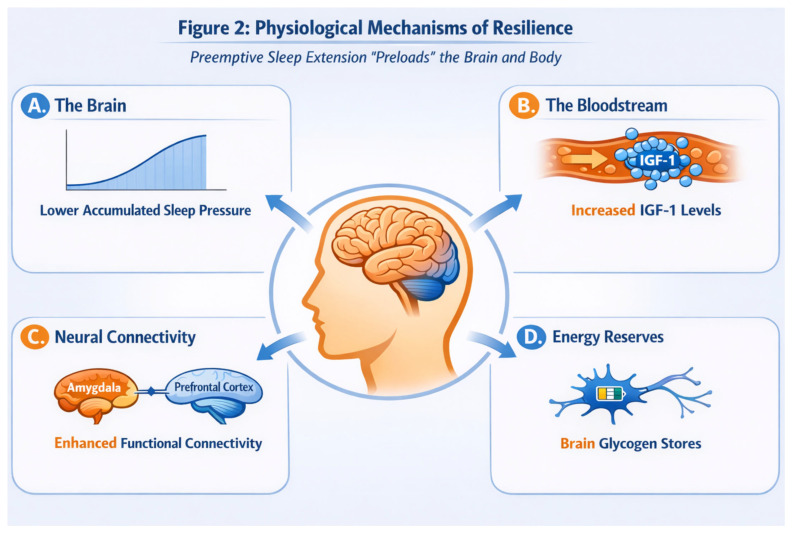
Physiological pathways supporting resilience after sleep banking (AI-generated figure created with ChatGPT, OpenAI, version GPT-4.1, accessed 20 December 2025).

Additionally, extra sleep might induce an upregulation of neuroprotective factors. The observed rise in IGF-1 after sleep extension supports the idea that neurotrophic or metabolic support pathways may be activated by abundant sleep [25]. IGF-1 and related growth factors can enhance synaptic plasticity and brain energy metabolism, potentially helping to sustain cognitive performance when challenged by sleep loss [27]. There is also speculation that sleep extension might bolster brain glycogen stores or other energy reserves that neurons can draw upon during prolonged wakefulness, although direct evidence in humans is limited [28]. (Figure 2. Physiological pathways supporting resilience after sleep banking (AI-generated figure created with ChatGPT, OpenAI, version GPT-4.1, accessed 20 December 2025).) Furthermore, sleep banking could influence other aspects of brain function. It may help maintain a more balanced hypothalamic–pituitary–adrenal (HPA) axis response under stress and might modulate neuromodulators such as dopamine and serotonin that regulate arousal and mood, though these possibilities have yet to be empirically confirmed. Notably, ensuring adequate sleep has been shown to normalize certain neural processes affected by sleep loss: for instance, resolving sleep debt can reduce hyperactivity in the amygdala and strengthen functional connectivity between the amygdala and prefrontal cortex, changes associated with better emotional regulation [29]. While direct data on these mechanisms in the context of sleep banking are still emerging, they highlight the multiple biological pathways through which extra sleep could confer resilience.

### 5.4. Practical Considerations and Feasibility

From a practical standpoint, the success of sleep banking depends not only on its physiological benefits but also on its feasibility and implementation. Encouraging individuals to get extra sleep is simple in concept but can be challenging due to work schedules, family responsibilities, or underlying insomnia. Real-world attempts at extending sleep show that the net gain in actual sleep may be modest. For example, in a free-living study, participants instructed to lengthen their time in bed did so by nearly an hour, but achieved only about 20 min of additional actual sleep on average, and their sleep efficiency slightly decreased [26]. This illustrates the difficulty of “forcing” extra sleep. Several factors may limit feasibility: individuals cannot easily fall asleep much earlier than their habitual bedtime if their circadian phase has not shifted accordingly; extended time in bed often results in more light sleep, more awakenings, and lower sleep efficiency, potentially limiting the restorative value per hour; those with greater pre-existing sleep debt may more readily extend sleep and show greater benefits, while well-rested individuals may have limited capacity to “bank” additional sleep; and multi-week extension protocols such as those employed by Mah et al. may not be practical before every anticipated sleep challenge. On the other hand, a positive finding is that significant issues with sleep inertia (morning grogginess after prolonged sleep) have not been prominently reported in sleep banking studies.

### 5.5. Applications in Specific Populations

In occupational settings, planners could incorporate extended sleep periods before predictable high-demand situations (like night shifts or long missions) as part of fatigue-risk management [30]. However, it should be noted that evidence supporting sleep banking in shift workers is currently weak: a systematic review found that the few studies available were of low quality and high risk of bias, making it hard to draw firm conclusions for shift-work populations [30]. Given that shift workers face combined homeostatic and circadian challenges, the laboratory evidence from non-shift-work samples may have limited generalizability. In athletic contexts, expert consensus guidelines explicitly recommend sleep extension to improve performance and recovery in elite athletes [31]. Similarly, military organizations might adopt sleep extension protocols during training or pre-deployment weeks to build a reserve of alertness and endurance before critical missions [19].

### 5.6. Methodological Considerations

The laboratory studies included in this review, while providing the most controlled evidence, share several methodological limitations that should be acknowledged. Most studies included 11–50 participants, limiting statistical power and generalizability. Samples were predominantly young (18–39 years), healthy, male adults without sleep disorders, leaving efficacy in older adults, women, or clinical populations unknown. Follow-up was typically limited to the immediate post-deprivation period, and long-term effects and safety of repeated sleep banking remain unstudied. Furthermore, benefits demonstrated under highly controlled laboratory conditions with fixed light, temperature, and activity may not translate to real-world settings with greater variability. These considerations apply equally to the laboratory evidence base; the critique of low quality evidence in shift workers [30] should be balanced against recognition that laboratory studies, while better controlled, have their own limitations.

## 6. Methods

We performed a literature search of PubMed, MEDLINE, and Embase databases for studies related to sleep extension and sleep banking published between January 2004 and August 2025. Search terms included combinations of “sleep extension”, “extended sleep”, “sleep banking”, “sleep debt”, “sleep deprivation”, and “performance”. We included peer-reviewed studies that investigated the effects of extended sleep prior to a period of sleep loss on cognitive, behavioral, or physiological outcomes. Both experimental laboratory studies (e.g., controlled sleep deprivation trials) and observational or epidemiological studies (including those involving shift workers or other real-world groups) were considered. Only human studies published in English were included. We excluded studies focusing solely on recovery sleep after deprivation, as the emphasis in our paper was on preemptive sleep gain rather than post hoc recovery.

The initial database search yielded several hundred records. After removal of duplicates, records were screened by title and abstract. The majority of exclusions at screening were due to studies examining only recovery sleep after deprivation rather than preemptive extension, studies without a subsequent sleep loss phase, animal studies, pediatric-only populations, non-English publications, and reviews or commentaries without primary data. Full-text articles were then assessed for eligibility, with exclusions for recovery sleep paradigms only, insufficient outcome data, and duplicate publications from the same dataset. Ultimately, 12 studies met inclusion criteria and are synthesized in this review (see Table 1): 7 primary experimental trials (total N ≈ 140 participants) and 4 secondary sources (systematic reviews, meta-analyses, expert consensus statements, and computational modeling studies). Skorucak et al. [32], although employing original EEG data from 35 participants in a crossover design, was classified as a secondary source because its primary objective was to validate and refine the two-process model of sleep regulation rather than to assess behavioral or cognitive outcomes of sleep banking per se. Studies examining circadian phase, shift timing, or chronotype were screened but were not included if they lacked a preemptive sleep extension intervention followed by sleep loss; the interaction between sleep extension and circadian biology represents an important gap in the current literature that we address in the Discussion.

Given the substantial heterogeneity in study designs (ranging from highly controlled laboratory sleep deprivation protocols to observational field studies), outcome measures (cognitive tests, physical performance, biomarkers), and populations (healthy young adults, athletes, military personnel, shift workers), a formal meta-analysis was not appropriate. The included studies employed different sleep extension durations (several nights to multiple weeks), different subsequent sleep challenges (partial restriction vs. total deprivation), and different primary outcome measures, precluding meaningful statistical pooling. A narrative synthesis allows for a nuanced discussion of converging evidence while acknowledging methodological differences.

Key data extracted from the selected studies included the study design (e.g., randomized controlled trial or observational), characteristics of sleep extension (duration and number of nights of extended sleep), the nature of the subsequent sleep loss (total deprivation vs. partial restriction), definition of the control condition (habitual sleep vs. fixed time in bed; how baseline sleep was monitored), outcome measures (cognitive performance tests, mood scales, physiological markers, health/safety indicators), and main findings. Throughout this review, “control condition” refers to participants maintaining their habitual sleep schedule or a standardized sleep opportunity (typically 7–8 h time in bed) during the baseline/pre-deprivation phase, as opposed to the “extended sleep” or “sleep banking” condition where time in bed was deliberately increased; the specific control parameters varied across studies and are detailed in Table 1. We gave precedence to controlled trials and systematic reviews for drawing conclusions, and for real-world applications, we noted evidence from field studies and any statistical associations reported in epidemiological research.

## 7. Conclusions and Future Directions

In summary, the current evidence from both laboratory experiments and field studies indicates that sleep banking is a viable strategy for mitigating the cognitive and physical deficits associated with subsequent sleep restriction. Obtaining extra sleep in advance of sleep loss confers measurable benefits in vigilance, cognitive performance, mood, and physical functioning, though it may not fully eliminate subjective fatigue. These findings support the concept that “banking” sleep can enhance resilience to sleep deprivation and may serve as a valuable complement to other sleep management strategies, but it is not a substitute for maintaining consistent, sufficient sleep on a regular basis.

### 7.1. Future Research Priorities

Several priorities should guide future research in this area. Studies should determine optimal protocols, including how many extra hours per night and for how many nights are ideal for different populations and anticipated sleep challenges. Establishing how long the benefits of banked sleep last and whether repeated banking maintains efficacy or shows diminishing returns is essential. Future work should also evaluate whether preemptive sleep can improve higher-order executive functions and complex decision-making, as current evidence for these domains is mixed.

Integrating circadian biology into sleep banking research represents a critical priority. Future studies should measure circadian phase markers such as dim-light melatonin onset and core body temperature minimum before and after sleep extension, assess chronotype using validated instruments and stratify analyses accordingly, test performance outcomes at multiple circadian phases to disentangle homeostatic and circadian contributions, compare sleep extension protocols that emphasize earlier bedtimes versus later wake times, and examine sleep banking in populations with known circadian disruption, including shift workers and transmeridian travelers.

Additionally, studies should rigorously characterize participants’ baseline sleep status using objective measures to distinguish between sleep debt repayment and creation of true reserve. Neuroimaging, molecular biomarkers, and polysomnography should be employed to investigate underlying mechanisms, including slow-wave activity dynamics and circadian modulation of restorative processes. Identifying who benefits most from sleep banking, considering genetic traits, chronotype, baseline sleep duration, and capacity to extend sleep, will be important for personalizing recommendations. There is also a pressing need for methodologically robust field studies, especially in shift work and operational settings, to validate laboratory findings in ecologically valid contexts. Finally, examining the safety and efficacy of repeated sleep banking across extended periods will be necessary to establish the long-term viability of this strategy.

### 7.2. Conclusions

Sleep banking represents a promising proactive strategy for enhancing resilience to anticipated sleep loss. While current evidence strongly supports benefits for vigilance and basic cognitive performance, important questions remain about higher-order cognition, circadian interactions, and real-world implementation. Integrating circadian biology into the study of sleep banking is essential for advancing both scientific understanding and practical application of this fatigue mitigation strategy.

## Figures and Tables

**Table 1:** Characteristics of Included Studies on Sleep Banking.

Study	N	Population	Design	Extension Protocol	Subsequent Sleep Loss	Control Condition	Primary Outcomes	Key Findings
Rupp et al., 2009, Sleep [11]	24	Healthy adults, 18–39 y	Parallel-group RCT	10 h TIB × 7 nights	7 nights of 3 h TIB	Habitual sleep (≈7.09 h)	PVT, MWT	Extended sleep reduced PVT lapses and improved MWT sleep latency during restriction
Arnal et al., 2015, Sleep [16]	14	Healthy men, 26–37 y	Crossover RCT	9.8 h TIB × 6 nights	38 h TSD	8.2 h TIB × 6 nights	PVT, MSLT, microsleeps, KSS	Sleep extension reduced PVT lapses and microsleeps during TSD; improved MSLT scores
Ritland et al., 2019, Sleep Med [19]	50	ROTC tactical athletes	RCT	≥8 h TIB (sleep extension)	None	Habitual sleep	PVT, SDMT, Flanker, TMT, SBJ, motivation	Sleep extension improved PVT, TMT, SBJ, motivation; persisted 4 days
Kamdar et al., 2004, Sleep Med [20]	15	College students, 18–23 y	Within-subject pre–post	Sleep extended ad libitum	None	Baseline (within-subject)	MSLT, PVT, POMS	MSLT increased significantly (*p* < 0.01); PVT improved; mood improved
Arnal et al., 2016, Med Sci Sports Exerc [21]	12	Healthy men	Crossover RCT	9.8 h TIB × 6 nights	34–37 h TSD	8.2 h TIB × 6 nights	Time to exhaustion, RPE, neuromuscular function	Sleep extension improved time to exhaustion (+8.1% after TSD); reduced RPE
Mah et al., 2011, Sleep [22]	11	Basketball players, 19.4 ± 1.4 y	Pre-post study	≥10 h TIB × 5–7 weeks	None	Baseline performance	Sprint time, shooting accuracy, PVT, ESS, POMS	Sprint improved (16.2→15.5 s); FT +9%, 3PT +9.2% (*p* < 0.001)
Vitale et al., 2019, Int J Sports Med [23]	—	Athletes	Narrative review	Sleep hygiene strategies	Athletic demands	N/A	Recovery, performance	Sleep extension improves reaction time, mood, sprint, accuracy
Chennaoui et al., 2016, Appl Physiol Nutr Metab [25]	14	Healthy men, 26–37 y	Crossover RCT	9.8 h TIB × 6 nights	24 h SD	8.2 h TIB × 6 nights	Free/total IGF-1, BDNF, GH, insulin, glucose	Sleep extension increased IGF-1 at baseline and during SD (*p* < 0.001)
Walsh et al., 2021, Br J Sports Med [31]	—	Athletes	Expert consensus	Individualized recommendations	Training/competition	N/A	Expert guidelines	Individualized approach preferred; research needed on sleep banking
Simpson et al., 2017, Scand J Med Sci Sports [33]	—	Athletes	Narrative review	Various protocols	Various	Various	Performance, cognition, health	Sleep extension improves reaction time, mood, sprint times, accuracy
Skorucak et al., 2018, Sleep [32]	35	Healthy adults	Crossover study	10 h TIB × 7 nights	Total sleep deprivation	6 h TIB × 7 nights	EEG SWA, SWE, REM sleep	Sleep restriction reduced REM; SWA consistent with two-process model
Bonnar et al., 2018, Sports Med [34]	218 (10 studies)	Athletes, 18–24 y	Systematic review	Various protocols	Training demands	Various	Athletic performance	Sleep extension had most beneficial effects on performance

Abbreviations: h = hours; IGF-1 = insulin-like growth factor-1; MSLT = Multiple Sleep Latency Test; N/A = not applicable; POMS = Profile of Mood States; PVT = Psychomotor Vigilance Task; RCT = randomized controlled trial; SD = sleep deprivation; SSS = Stanford Sleepiness Scale; TIB = time in bed; TSD = total sleep deprivation; TST = total sleep time; y = years.

## Data Availability

Data sharing is not applicable to this article, as it is a narrative review and no new, original data were created. All information synthesized in this review is derived from previously published studies, which are publicly available and cited in the References section.

## Glossary

- Chronotype: An individual’s natural preference for sleep–wake timing, typically characterized on a spectrum from “morning types” (early to bed, early to rise) to “evening types” (late to bed, late to rise). Chronotype is influenced by genetic, environmental, and age-related factors.
- Circadian Rhythm (Process C): An approximately 24 h biological rhythm, generated by the suprachiasmatic nucleus of the hypothalamus, that modulates sleep propensity, alertness, and physiological functions independently of prior sleep history.
- Homeostatic Sleep Pressure (Process S): The biological drive for sleep that accumulates during wakefulness and dissipates during sleep. Entering a period of sleep loss with lower accumulated sleep pressure is a proposed mechanism for sleep banking’s benefits.
- Insulin-like Growth Factor 1 (IGF-1): A hormone involved in neuroplasticity, metabolism, and tissue repair. One study noted that sleep extension significantly increased IGF-1 levels.
- Maintenance of Wakefulness Test (MWT): A test used to measure an individual’s objective ability to remain awake.
- Microsleeps: Brief, involuntary, EEG-confirmed episodes of sleep that occur during wakefulness.
- Psychomotor Vigilance Task (PVT): A test measuring sustained attention by recording reaction times to a stimulus. “Lapses” on this test (slow or missed responses) are a key indicator of cognitive impairment from sleep loss.
- Sleep Banking (or Preemptive Sleep Extension): A proactive strategy that involves obtaining extra sleep in advance of an anticipated period of sleep restriction or total sleep deprivation.
- Sleep Debt: The cumulative effect of chronic sleep insufficiency, representing the difference between the amount of sleep needed and the amount obtained.
- Sleep Inertia: A state of grogginess and reduced cognitive performance that can occur immediately after waking up.
- Sleep Restriction: A period where sleep duration is curtailed (e.g., 3 h in bed nightly), as distinct from total sleep deprivation.
- Social Jetlag: The discrepancy between an individual’s biological clock timing and socially imposed sleep schedules (e.g., work or school start times), often resulting in chronic circadian misalignment and sleep curtailment.
- Total Sleep Deprivation: A period of complete, continuous wakefulness, such as 38 h or an entire night.
- Two-Process Model: A theoretical framework proposing that sleep timing and intensity are governed by the interaction of homeostatic (Process S) and circadian (Process C) processes.
